# SHARE – workpackage 5: evidence based recommendations for diagnosis and treatment of the antiphospholipid syndrome

**DOI:** 10.1186/1546-0096-12-S1-P111

**Published:** 2014-09-17

**Authors:** Noortje Groot, Nienke de Graeff, Tadej Avcin, Brigitte Bader-Meunier, Paul Brogan, Pavla Dolezalova, Brian Feldman, Isabelle Kone-Paut, Pekka Lahdenne, Alberto Martini, Liza McCann, Seza Ozen, Clarissa Pilkington, Angelo Ravelli, Annet van Royen, Bas Vastert, Nico Wulffraat, Sylvia Kamphuis, Michael Beresford

**Affiliations:** 1 Alder Hey Children's Hospital, Liverpool, UK; 2 Erasmus MC-Sophia, Rotterdam, Netherlands; 3 University Medical Centre Utrecht, Utrecht, Netherlands; 4 University Medical Centre, Ljubljana, Slovenia; 5 Necker Hospital, Paris, France; 6 Great Ormond Street Hospital, London, UK; 7 General University Hospital, Prague, Czech Republic; 8 Sick Kids Hospital, Toronto, Canada; 9 CHU de Bigetre, Paris, France; 10 Children's Hospital of Helsinki and Uusimaa, Helsinki, Finland; 11 Gaslini Children's Hospital, Genova, Italy; 12 Hacettepe University Children's Hospital, Ankara, Turkey

## Introduction

Antiphospholipid syndrome (APS), either primary or secondary to other paediatric rheumatic diseases, is rare in children, but it can lead to significant morbidity. Evidence-based guidelines are sparse and management is mostly based on physician’s experience. Consequently, treatment regimens differ throughout Europe. In 2012, a European initiative called SHARE (Single Hub and Access point for paediatric Rheumatology in Europe) was launched to optimize and disseminate diagnostic and management regimens in Europe for children and young adults with rheumatic diseases such as APS.

## Objectives

To provide evidence based recommendations for diagnosis and treatment of APS.

## Methods

Evidence based recommendations were developed using the European League Against Rheumatism (EULAR) standard operating procedure. An expert committee was instituted, consisting of paediatric rheumatologists from across Europe with expertise in APS. The expert committee defined search terms for the systematic literature review, which was performed in summer 2013. Two independent experts scored articles for validity and level of evidence. Recommendations derived from the literature were evaluated by an online survey. Those with less than 80% agreement during the online survey were reformulated. Subsequently, all recommendations were discussed at a consensus meeting using the nominal group technique[1]. Recommendations were accepted if more than 80% agreement was reached.

## Results

The literature search yielded 1463 articles, of which 15 ( all relating to diagnosis only, none were relevant for treatment) were considered relevant and therefore scored for validity and level of evidence. Only 8 articles were deemed valid and were used in the formulation of the recommendations. In view of paucity of paediatric-specific data, the majority of proposed recommendations were developed based on adult-derived literature or expert opinion. Four recommendations for diagnosis and 2 for treatment were suggested in the online survey. During the consensus meeting, recommendations based on expert opinion were added. Three recommendations for diagnosis and 6 for treatment were accepted with more than 80% agreement after the consensus meeting. Table 1 summarizes the categories of recommendations.

**Table 1:** 

Recommendations regarding:	Number
Diagnosis	
Classification	2
Laboratory features	2

Treatment:	
Preventive	1
For venous thrombotic events	2
For arterial thrombotic events	2

## Conclusion

The SHARE initiative provides recommendations for diagnosis and treatment for APS and thereby facilitates improvement and uniformity of care throughout Europe. Currently, a similar process is going on to add additional guidelines including those on holistic care for PRD patients. As a final result, SHARE will provide standards of minimal care for different PRDs, including APS.

## Disclosure of interest

None declared.

